# ‘Skin Trade’: Genealogy of Anti-ageing ‘Whiteness Therapy’ in Colonial Medicine

**DOI:** 10.1007/s12376-014-0089-8

**Published:** 2014-03-21

**Authors:** Amina Mire

**Affiliations:** Department of Sociology and Anthropology, Carleton University, 1125 Colonel By Drive, B750 Loeb Building, Ottawa, ON K1S 5B6 Canada

**Keywords:** Anti-ageing, Do-it-yourself, Biomedicalization of ageing, Racial degeneration, Whiteness therapy, Tropical clothing

## Abstract

This article investigates the extent to which the emerging trend of do-it-yourself anti-ageing skin-whitening products represents a re-articulation of Western colonial concerns with environmental pollution and racial degeneracy into concern with gendered vulnerability. This emerging market is a multibillion dollar industry anchored in the USA, but expanding globally. Do-it-yourself anti-ageing skin-whitening products purport to address the needs of those looking to fight the visible signs of ageing, often promising to remove hyper-pigmented age spots from women’s skin, and replace it with ageless skin, free from pigmentation. In order to contextualize the investigation of do-it-yourself anti-ageing skin-whitening practice and discourse, this article draws from the literature in colonial commodity culture, colonial tropical medicine, the contemporary anti-ageing discourse, and advertisements for anti-ageing skin-whitening products. First, it argues that the framing of the biomedicalization of ageing as a pigmentation problem caused by deteriorating environmental conditions and unhealthy lifestyle draws tacitly from European colonial concerns with the European body’s susceptibility to tropical diseases, pigmentation disorders, and racial degeneration. Second, the article argues that the rise of do-it-yourself anti-ageing skin-whitening commodities that promise to whiten, brighten, and purify the ageing skin of women and frames the visible signs of ageing in terms of pigmentation pathology.

## Racial (Re)-Degeneration: Colonial Medicine and the ‘Therapeutic Effects of Whiteness’

According to historians, European colonial medicine played a central role in the conceptualization of the dark skin tones of colonized people as signs of racial degeneration from the late seventeenth century until the end of the colonial era, in the early twentieth century (Eze 1997; Johnson 2008). The historical literature in European colonial medicine shows repeated attempts to make direct links between climate difference and difference in skin colour, as well as attempts to link dark skin tones with disease and racial degeneration. In particular, literature in European colonial medicine makes causal links between tropical climate and racial degeneration, disease, and decline (Ahmed 1998; Johnson 2008, 2009). Climate aetiology has also been used to generate new moral, philosophical and political paradigms, theories, and concepts through which a new European identity as white and civilized was constituted, regulated, and protected from pigmentation diseases and racial degeneration. In this way, colonial medicine and climate discourse became critical sites through which the **‘**truth’ (Foucault 2003; Stoler 2000, 2002) of European corporeal health and moral superiority came to be discursively produced, normalized, and transmitted (Ahmed 1998; Benthien 2002; Eze 1997; Johnson 2008).

In her work, *Skin: On the Cultural Border Between Self and the World*, Benthien (2002) traces how in the late seventeenth- and eighteenth-century German, French, and other European philologists and natural historians mobilized the pigmented skin as a marker of difference between civilized, white Europeans and the uncivilized, degenerate, non-white, colonized races (Benthien 2002, p. 145). According to Benthien, late seventeenth-century German philosophers and natural historians argued that tropical heat was the cause of the darkening of skin colour. In addition, they argued that if the tropical heat was the cause of the darkening of skin colour, and so the disease and decline of non-white ‘races’, the colder European climate and landscape must therefore be the cause of European health, vigour, enterprise, discipline, whiteness, and superior civilization. According to this theory, the white European skin became a symbolically visible site on which to territorialize and embody the new European racial identity of whiteness. However, representing the colder European climate, the direct source of European health, vigour, and whiteness presented new challenges in the context of increased European competition for the colonial scramble for Africa and other tropical zones.

### Tropical Spaces, ‘Racial Degeneration’, and Pathology

Thus, in the age of European colonial expansionism, tropical spaces were represented as repositories of immense wealth but also as sites of disease and racial degeneration (Johnson 2008). As a result, how to preserve European health in the tropical zones became a key strategy to the European colonial rule of the tropics. European colonial interests and anxiety of tropical diseases also heightened European curiosity to know and master the causes of black skin and blackness of the African body: its physiology, texture, and its functioning (Benthien 2002; Eze 1997, p. 71). For instance, in 1735, the German anatomist Zedler claimed that the dark skin tone of the ‘Moorish’ (i.e. black) skin was caused by a faulty morphological structure below the upper membrane, underneath the skin, which he termed ‘Malphagi membrane’ (Benthien 2002, p. 148). Zedler opined that, as result of its faulty physiology, the Malphagi membrane in Moorish skin was a very thin pad with countless holes inside which black pigments could accumulate, thereby making the Moorish skin appear dark and swarthy (Benthien 2002, p. 148).

In this way, Zedler was able to offer a deeper medical account of causes of dark skin. Zedler offered an early version of a medical construction of dark skin as a sign of corporeal pathology and racial degeneracy. Zedler’s analysis of the hyper-pigmented dark skin represents a late seventeenth-century articulation of European colonial tropical medicine and racial hygiene. In turn, the dark-skinned African’s capacity to accumulate black pigment was emphasized to make discursive distinctions between the pigmented, potentially dirty, and biologically degenerate non-white races from the pigment-free, clean, physiologically efficient, evolutionarily superior European white colonial race.

### Metaphysical Dualism and ‘Whiteness’ in the Age of Empire

Even philosophers of the period could not resist using the literature from colonial medicine to construct their own versions of racial epistemology. In turn, new racial epistemology was used to reinforce certain supposedly fundamental racial differences between Europeans and non-Europeans. Thus, according to Kant:

For now we know: human blood becomes black only because it is overloaded with phlogiston (as can be seen from the underside of clotted blood). Now the strong smell of the Negroes, which cannot be avoided by any kind of hygiene, in itself gives reason to suppose that their skin is removing a large amount of phlogiston from the blood and that nature must have organized this skin in such a way that the blood can be dephlogistonized by it to a much greater degree than occurs in us, in whom that is for the most part the business of the lung. (Kant, quoted in Benthien 2002, p. 151)

The above quote shows Kant’s willingness to suspend a deep commitment to universal ahistorical and metaphysical dualism of the mind and body in favour of empirically grounded, superficial difference in skin colour the cause of supposed African racial inferiority. However, Kant’s turn on the European embodiment of whiteness did not mean he completely abandoned his universal metaphysical philosophy. Kant’s racial epistemology tacitly allows potential substitution of European universal identity from objective, metaphysical philosophy to subjective, epidermalized whiteness. Furthermore, Kant’s racial epistemology suggests that the European white body has the capacity to suffer from pigmentation problems, corporeal pollution, and racial degeneracy if exposed to the extreme tropical climate. Hence, it is not inconsequential that as European colonial competition for Africa and other tropical zones intensified, European colonial medicine turned its primary attention to assessing, containing, and ameliorating the threats that tropical diseases posed to ‘Anglo-Saxon health’ (Johnson 2009).

Thus, in the late nineteenth-century British imperial and colonial context, the domain of tropical medicine became as much concerned with ‘the preservation of white European health in the tropical climate’ as spreading and justifying British colonial rule in the name of a ‘civilizing mission’ born of the ‘White Man’s Burden’ (Johnson 2009; McClintock 1995). As a result, British fear and anxiety of the potential diffusion of pigmentation pathologies from tropical colonial spaces and bodies to European bodies and the European continent became permanent features of the production, dissemination, and use of knowledge in colonial medicine and racial hygiene (Johnson 2009; Stoler 2002).

### Commodity Culture: Tropical Clothing and Racial Hygiene

One of the strategies adopted by British and other European colonial medical experts was to design clothes capable of shielding the European body from the harmful effects of tropical climates. While the experts sometimes disagreed which fabric provided best protection: cotton, linen, wool, silk, or some combination; thereof, they agreed that it should be that which most efficiently mimicked the protective ‘cutaneous qualities’ of the ‘black’ skin—and not the native ways or manner of dress (Brenner and Hearing 2008). Ironically, the pigmented skin which was cast as degenerate and the site of pathology simultaneously provided the inspiration for the ‘suitable clothing that the modern man of the north will be enabled to conquer the exigencies of tropical life’ (Johnson 2009).

In 1907, James Cantlie, lecturer in surgery at the London School of Tropical Medicine and cofounding editor of the *Journal of Tropical Medicine*, published an article addressing the importance of European clothing in relation to African dress and cutaneous qualities of skin. (Johnson 2009)

In a move that seems to repudiate the very founding theory of tropical medicine and racial hygiene, Cantlie cited recent advances in ‘tropical clothing’ to support the belief that the pigment in black skin has a protective shield which helps the black man to adapt to his environment (Brenner and Hearing 2008). What is of particular interest here is that it seems that the protective quality of the black skin can be appropriated, transformed and put in service in the form of ‘white’ linen, or cotton to provide similar protective shielding to the white Europeans in the tropics. In doing so, Cantlie mediates and attenuates the unsayable but protective quality of the black skin by symbolically transforming it and displacing it into the tropical ‘white skin’ (i.e. tropical clothing designed to protect to unprotected white skin). The fabric therefore becomes a surrogate for missing pigment, as Cantlie notes, ‘the skin of the white man is unprovided with the protective pigment which adapts the coloured man to his environment’ (Johnson 2009). Cantlie’s slippage is critically important because, as McClintock notes, white colonial linen became one of the potent visual symbols of the British colonial rule and imperial domination of non-white peoples and continents (McClintock 1995; Anderson 1996). Hence, through this clever displacement, white Europeans could fashion the cutaneous qualities of black skin without compromising the colour-coded differences between white Europeans and tropical blacks. In addition to the basic utility of mimicking the protective advantage of the black tropical skin, white colonial linen functioned as a symbolic boundary object through which to regulate contact between colonized, non-white communities and the colonizing, ruling European classes (McClintock 1995; Anderson 1996).

White colonial linen also tacitly regulated the sexual purity of European women the tropics. The perceived threat of dangerous sexual liaisons between members of the non-white others and Europeans was also expressed through the practice of racial hygiene. Such liaisons, if not carefully regulated could compromise racial purity and the health of the white European body (McClintock 1995; Stoler 2002; Anderson 1996). Thus, the fear of contracting the contagion of the black skin became one of the key concerns of tropical medicine. Hence, as early as in the seventeenth-century, German natural historian and philologist, Herder, took a keen interest in ascertaining the ‘colour’ of black men’s semen fluids and brain tissues. Upon presumably conducting dissections on black African men’s bodies and by obtaining samples of semen fluids and brain tissues from them, Herder informed his anxious European readers that neither the blood nor the brain nor the semen fluids of the negroes was black (Benthien 2002, p. 148). Herder’s ambivalent reference that the African male’s body fluids contained no blackness registers the European colonial anxiety of not only racial degeneration from the hot tropical climate, but also fear of interracial miscegenation between Europeans and the dark-skinned colonized people of the tropics (Gilman 1985). Blumenbach, another seventeenth-century German natural historian, advanced a very influential environmentalist theory of racial degeneration. In his 1775 monograph, *On the Natural Varieties of Mankind*, Blumenbach claimed climate difference was the primary cause of visible colour gradations and other visible differences between populations living in different parts of the world. In addition, he claimed that original humans had white skin and European features—what he coined to be a ‘Caucasian’ race. He further asserted that brown, black, and yellow hued skins represented the various stages of environmentally induced ‘degeneration’:

Caucasian Variety. I have taken the name from Mount Caucasus, both because of its neighborhood, and especially the southern slope, produces the most beautiful race of men, I mean the Georgians; and because all the physiological reasons converge to this, that in this region, if anywhere, it seems we ought with greatest probability to place the autochthones of mankind… That stock plays…the most beautiful form of the skull, from which, as from a mean and primeval type, the others diverge…. Besides, it is white in color, which we may fairly assume to be the primitive color of mankind, since…it is very easy to degenerate into brown, but very much more difficult for dark to become white. (Blumenbach, *On the Origin of the Natural Varieties of Mankind,* 1775, quoted in Jacobson 1998, p. 1)

Blumenbach’s ‘degeneration’ became a powerful ideological imperative through which the symbolic embodiment of whiteness came to be constructed to mark spatial and moral boundaries between colonized natives and European colonial classes (Gilman 1985). It also has enduring relevance. First, as already demonstrated, colonial medicine and racial hygiene reinforced the European civilizing mission of ‘the white man’s moral burden’ to bring, by diffusion, the superior benefits of whiteness to the non-white colonized races of the other (Blaut 1992; Butchart 1998). Second, it facilitated the phenomenal rise of the European colonial commodity culture (Johnson 2008; McClintock 1995). That is, medical discourses of ‘racial degeneration’ and ‘corporeal hygiene’ were used in promotions as diverse as manual guides to proper nutrition and medical textbooks, to selling soap and clothing. Therefore, consumption of health-promoting commodities became another site through which the contradictory discourses of colonial exploitation and imperial rule could be defused, mediated, and normalized (McClintock 1995). McClintock offers an excellent account of nineteenth-century context advertisements for commodities, such as soap in a Victorian British imperial society and in the colonies. She argues that in Victorian commodity culture, cheap commodity items such as soap were imbued with ‘magical’ power to cleanse and ‘regenerate’ non-white colonized bodies while safeguarding the health and vigour of the white European colonizers both at home and in the colonial spaces.

McClintock suggests that soap, signifier of imperial prowess and ‘racial regeneration’, became popular not at the height of colonial vigour and control but at the moment in nineteenth-century British culture when its colonial hegemony began to be contested by anti-colonial struggles and fierce colonial competition from other European nations:

Soap did not flourish when imperial ebullience was at its peak. It emerged commercially during an era of impending crisis and social calamity, serving to preserve, through fetish ritual, the uncertain boundaries of class, gender and race identity in a social order felt to be threatened by fetid effluvia of the slums, the belching smoke of industry, social agitation, economic upheaval, imperial competition and anticolonial resistance. Soap offered the promise of spiritual salvation and regeneration through commodity consumption, a regime of domestic hygiene that could restore the threatened potency of the imperial body politic and the race. (McClintock 1995, p. 211)

Analysis of nineteenth-century advertisements for the colonial commodity of whiteness, such as linen, cotton, silk, wool and soap, and an analysis of the broader discourse of tropical medicine and racial hygiene, can help this author to analyse social, ethical, and economic imperatives that support and sustain contemporary and globalizing trends of anti-ageing skin-whitening commodities, which promise to ‘restore’, ‘regenerate, and ‘purify’ the ageing skins of women globally, and middle aged white women in particular. These products promise ‘ageless beauty’ and glowing white skin to consumers by removing visible signs of ageing; that is, age spots, photo-ageing, and hyper-pigmentation. This author contends that anti-ageing skin-whitening tacitly borrowed and re-articulated, albeit in different historical and material contexts, Western colonial anxiety and fear of racial degeneracy and gendered vulnerability. One significant difference between the colonial medicine and contemporary anti-ageing discourses is that women are the primary target for anti-ageing promotion, whereas colonial medicine paid attention to the health needs of both the colonial male officers and their families. In this work, I focus primarily on skin-whitening products promoted as anti-ageing skin-whiteners. However, it is pertinent to briefly address the centrality of gender to the aggressive marketing of anti-ageing to women. For example, in *Don’t Touch Me (I’m Electric): On Gender and Sensation in Modernity*, Henning (1999) analysed how anti-ageing promotion draws from deeply entrenched, dominant Western medical discourses of the colonial era:

Apparently I am at risk. The stresses of a busy urban lifestyle, pollution and holes in the ozone layer are undermining the protective role of that important bodily organ – my skin. Unprotected, I start to display the symptoms of an illness called ageing. Happily, though, there are skin creams which can restore the protective powers of my skin. Scientific research has produced a new ‘skin technology’ which can save me. (Henning 1999, p. 17)

Henning’s analysis helps my current investigation in how discursive representation of women’s bodies as uniquely vulnerable to urban pollution, stress, and premature ageing, facilitates aggressive marketing of anti-ageing products to them. It is one of the claims of this author that the biomedicalization of ageing is key to a broader understanding contemporary dynamics of science, ecology, gender, race, class, and ageism in the emerging anti-ageing discourse and practice. This paper does not seek to address questions raised in the domain of ‘anti-ageing medicine’ (Petersen and Seear 2009). Instead, it examines promotional material for unregulated do-it-yourself products which link anti-ageing to skin whitening. The aim is to examine the broader social, political, health, and ethical implications of emerging anti-ageing skin-whitening. Since skin-whitening is in this context linked with anti-ageing, skin-whitening will be examined as a form of do-it-yourself ‘whiteness therapy’. Approaching anti-ageing skin-whitening in terms of fighting ‘pigmentation’ problems of ageing is a useful way to examine the extent to which anti-ageing skin-whitening represents at once a contemporary transcendence and re-articulation of colonial ‘tropical’ medicine. Comparative analysis of the rise of tropical medicine, racial hygiene, their corollary colonial commodity culture, and the emerging phenomenon of anti-ageing skin whitening is useful since both phenomena emerged as a result of real and imagined fears and anxieties pertaining to so-called environmentally caused diseases, degeneration, and decline. This comparative analytical strategy allows the author to link contemporary concern with pigmentation and ageing and colonial concern with pigmentation and racial degeneration. Contemporary skin-whitening can be understood as a form of anti-ageing whiteness therapy sold as a cure for pigmentation problems, wherein the skin-whitening anti-ageing industry relies on the notion of whiteness as a transferable therapeutic commodity that can be bought a sold. Through this process of commodification, anti-ageing skin-whitening therapy can be historically and symbolically disentangled from the discourse of colonial medicine by simultaneously appealing to local and particular concerns of women and the globalizing symbolic value of whiteness. Therefore, anti-ageing skin-whitening practice and discourse is grounded both in locality, particularity and in the universal notion of modernity. For instance, when marketing to Asian women, advertisements for anti-ageing skin-whitening products often stress both traditional Asian aesthetic preferences for light skin alongside an investment in deracialized universal beauty (Gosai 2010; Jesús 2005; Osuri 2008). Contemporary anti-ageing skin-whitening is dynamic, complex, and globalizing. The contemporary globalizing effect of whiteness can be gleaned from Bollywood films to corporate advertisements, fashion magazines, and online promotions—sites through which anti-ageing whiteness is discursively produced and circulated across national and international boundaries (Gosai 2010; Jesús 2005; Osuri 2008). Whereas colonial biomedicine was concerned with pigmentation as a form of racial degeneration in white subjects, contemporary concern with pigmentation transcends colonial boundaries of race, class, and gender, since, now, whites and non-whites can equally suffer from ‘pigmentation problems’. Consequently, contemporary pigmentation pathology is framed overwhelmingly as a women’s problem. However, as in colonial times, pigmentation problems have been understood as environmentally induced ailments. Therefore, anti-ageing whiteness therapy seeks to address the vulnerability of women to environmentally induced hyper-pigmentation (Henning 1999).

The following section seeks to address the extent to which contemporary concerns regarding climate change, the thinning of the Earth’s ozone layer, and urban pollution facilitate the emerging ‘do-it-yourself’ anti-ageing skin-whitening commodity culture—which targets not exclusively but overwhelmingly the white female body as particularly amenable to anti-ageing skin-whitening interventions. That is, the following section will investigate how advertisements for anti-ageing with skin-whitening properties promise to restore the youthful appearance of female consumers by ‘purging’, ‘suppressing’, and ‘cleansing’ unwanted pigmentation accumulation caused by an ‘accelerated’ or ‘premature’ ageing process.

## ‘New-Skin’: Anti-ageing Skin-Whitening ‘Therapy’

The conflation of benign but visible ageing symptoms—sometimes called age spots, photo-ageing, or hyper-pigmentation—with medically legitimate pigmentation disorders is evident not only in popular advertisements for anti-ageing products but also in scholarly papers published in peer-reviewed medical journals (Seiberg et al. 2000; Villarama and Mailbach 2005). In this way, expensive products that promulgate their scientific potency to ‘cure’, ‘reverse’, and ‘halt’ both the visible signs of ageing and the biological process of ageing, collapse their curative qualities with whitening effects. Promotional materials for anti-ageing skin-whitening products often promise to remove unwanted melanin from the user’s skin, reverse the ageing process, and keep the consumer’s skin ageless, radiant, and glowing. The following advertisement for a skin-whitening serum called, ‘Ageless: total skin lightening serum’ is an example of how anti-ageing skin whitening conflates anti-ageing with whiteness therapy (New Beauty SPA+ 2013). In addition to the anti-ageing skin-whitening serum company’s website, imageskincare.com, advertisement for this product line was placed in the first issue of a high-end beauty and spa magazine (New Beauty SPA+ 2013, p. 89). Image Skincare products ‘are all about delivering results, not just a feeling’ (New Beauty SPA+ 2013, p. 89), according to their two page spread in the inaugural issue of New Beauty Spa+. The ‘results’ that CEO Janna Ronert references include the power to reverse the ageing process simply by reversing the aesthetic symptoms of ageing and by whitening already existing visible signs of ageing to illustrate this point, their ‘Ageless the MAX™ Serum’ is a:

Revolutionary day and night serum that uses peptides, apple stem cells and botanicals to plum the skin, repair cell damage and dramatically reduce fine lines and wrinkles. (New Beauty SPA+ 2013, p. 89)

### An Expert’s Do-it-Yourself Guide to ‘Ageless Beauty’ and ‘Miracle Whiteness’

What is particularly intriguing about anti-ageing skin-whitening advertisements such as the one quoted above is the suggestion that benign but visible signs of ageing, such as fine lines and wrinkles, can be mitigated through skin whitening. It is in the context of making a direct association between anti-ageing and skin whitening that the broader social, health, ethical, and legal implications of do-it-yourself anti-ageing skin-whitening must be critically examined to reveal its potential capacity to reinforce the racialization and biomedicalization of women through its appeal to globalized notions of whiteness as a form of universalised wellness. It is pertinent to stress that do-it-yourself anti-ageing is mediated by the powerful testimony of medical ‘experts’ with vested interests in an emerging and highly lucrative enterprise. Some medical doctors use their academic credentials as part of their trademark to give extra scientific weight to their anti-ageing brand. Here is an example of one such brand: ‘time arrest^®^ crème de LUXE’. This supposedly time arresting anti-ageing cream is promoted as a non-prescription cosmetic, despite the fact that its advertising claims ought to render it a drug according to FDA drug law:

Introducing time arrest^®^ crème de LUXE, Dr. Brandt’s luxuriously rich cream that fights the signs of aging on many levels. This unique multi-faceted formula is enriched with grape stem cells, pearl silanols, tourmaline crystals, and AGE REVERSING PLATINUM TECHNOLOGY to capture the full spectrum of a perfect luminous, glowing complexion. (New Beauty 2010, p. 65)

It is noteworthy that this advertising example also makes a direct link between anti-ageing and a ‘glowing complexion’. What is more, as if using his professional medical credentials in the branding of this anti-ageing product was not enough, ‘age-reversing platinum technology’ was written in capital letters in order to highlight what Mire (2005) calls symbolic illustration of ‘the technological prowess of advanced skin-whitening biotechnology’ (Mire 2005, p. 10). Aggressive use of scientific rhetoric in most anti-ageing skin-whitening promotions in popular magazines and on the internet reveals that anti-ageing is an emerging, multifaceted domain with strong economic, scientific, and social investments (Mire 2012; Petersen and Seear 2009). As already stated in this paper, growing interest in anti-ageing knowledge is reflected in leading, peer-reviewed, medical journals, and popular, commercially oriented, corporate websites and print media. It is in this context that both popular and scholarly reports on the latest patented or soon to be patented ‘breakthrough’ anti-ageing and skin-whitening *actives*—agents with medicinal value—and the concomitant discourse of unlocking the ‘genetic code’ of the pigmented skin in order to minimize, suppress, or eliminate visible signs of melanin must be comprehended as new regime of what Rose calls *bioeconomics* (Rose 2007).

### Shifts in Health Consumerism: ‘Self-responsibility’ and the Biomedicalization of Ageing

In *The Politics of Life Itself: Biomedicine, Power, and Subjectivity in the Twenty*-*First Century*, Rose (2007) outlines the bioeconomics of health as a one of the key features of the current discourse and practice of biomedicine, marked by the proliferation of breakthrough innovations in molecular-biology- and genetics-based research. These developments are qualitatively different from older biomedical models, notably Foucault’s analysis of the rise of nineteenth-century clinical medicine dominated by experts. Instead, today’s patients and potential patients can and do play significant roles in shaping the research agendas, development procedures, access to and use of biomedical knowledge and services (Foucault 1994; Rose 2007, p. 22). According to Rose (2007), new breakthroughs in molecular-biology- and genetics-based biomedicine in the context of the globalization of mass media—including electronic media’s ‘popularization of medical knowledge’—have given rise to a new ‘ethicopolitics’ of ‘active citizenship’. In this new ‘ethicopolitics’, real and potential patients seek access to customized and evidence-based medical care and medical knowledge, and in so doing are transformed from ‘passive recipients of expertly directed medical care to active consumers of health’ (Rose 2007, pp. 22–27). Critical to this shift in health consumerism from passive patients to active citizenship holders is the ethical requirement of ‘self-responsibility’ (Clarke et al. 2003; Gerlach et al. 2011; Rose 2007). As the material examined in this paper has demonstrated, the emergent anti-ageing skin-whitening market plays directly into this notion of self-directed health consumerism. However, when it comes to the do-it-yourself anti-ageing material examined herein, the concept of self-responsibility operates in a context of high-technology language, expert direction, and complete unregulation, where commodities are marketed directly to consumers. With respect to the anti-ageing skin-whitening market, what is not fully captured in Rose’s argument is that what is being sold is not evidence-based medical knowledge, but dreams and fantasies of immortality and ageless beauty. Moreover, in do-it-yourself anti-ageing skin-whitening commercial promotional material, one’s ‘personal choice’ and ‘self-responsibility’ to be ageless, attractive, youthful-looking specifically by selecting and consuming appropriate products is shaped by the expert opinions of aggressively enterprising scientific entrepreneurs (Khan 2007). As a result, individual agency in choosing to practice an anti-ageing lifestyle operates in a broader social context in which those who choose freely not to practice anti-ageing consumerism—such as anti-ageing skin-whitening—or those who cannot afford to purchase these expensive commodities are discursively erased or stigmatized (Mire 2012; Spindler and Streubel 2009; Winterich 2007). Therefore, the biomedicalization of ageing plays a significant role in emerging anti-ageing market.

From the covers of glossy fashion magazines, the first series of high-end anti-ageing magazine, *New Beauty*, and plethora of online sites run by leading cosmetics corporations and biotechnology firms, aggressive marketing directives which encourage women to self-diagnose for imperfections and peruse expert tips and cosmetics products designed specifically to ‘cure’ various imperfections and diseases are sewn. While consumers can indeed make their own choice whether or not to use any give product, it is important to bear in mind that consumer-direct advertisements for anti-ageing skin-whitening products that can penetrate the body and transform it into young looking appearance at any age point to flaws in the consumer choice rationale when they promulgate metaphors of scientific mastery over the human body, which the average consumer may not be able to evaluate critically within the pervasive biomedicalization of ageing discourse.

In this context, expert driven promotion of high-end anti-ageing skin-whitening reinforces self-surveillance and self-medicalization through such techniques as ‘self-help’ brochures which encourage consumers to identify their own imperfections and seek remedies through cosmetics, such as ‘curing’ age spots with high-technology-based skin-whitening creams and serums. In this way, systematic pathologization of women’s bodies and skin as vulnerable to premature ageing and similar ‘pigmentation disorders’ enable and reinforce the discursive production and normalization of anti-ageing practice. For these reasons, this article investigates how emerging anti-ageing skin whitening has been reinforced with the discursive construction of the successful anti-ageing skin-whitening consumer as a ‘younger looking’ female with ‘radiant’, white skin. This paper shows that anti-ageing skin whitening also requires the discursive construction of women with visible signs of ageing, by labelling visible signs of ageing as markers of racial degeneration and gendered vulnerabilities. The discursive conflation of ‘whiteness therapy’ with anti-ageing so dominant in promotional material for the products, the association of white skin with heath and pigmented skin with disease, decline and degeneracy is repeated regardless of context, ethnicity, and skin colour of the intended consumer.

Additionally, even when the product in question is not high end but relatively cheap in comparison with its brand name equivalent, advertisers make certain that the supposed miracles of natural products are reinforced with patented high-technology innovations in anti-ageing skin whitening. The following advertisement for a popular anti-ageing skin-whitening brand, which appeared in New Beauty Magazine (New Beauty 2010), featured Hollywood actress Cate Blanchett. Her blonde hair is pulled back and her ethereal white face and arms provide the bottle of ‘SK-II: Facial Treatment Essence’. The bottle is transparent and shows the white cream inside. The cap of the bottle is brilliant silver and the caption reads:

“9 years on, and I’m still happy I found SK-II. There is nothing else like it”. Cate Blanchett. The “Miracle Water” with Natural Derived Pitera™ for clear, glowing skin. Discovered in a sake brewery in Japan – when people noticed older workers still had remarkably youthful-looking hands – this seemingly “age-defying” phenomenon sparked scientific research, which led to the miracle ingredient, SK-II Pitera:™ an exclusive blend of vitamins, minerals, organic and amino acids that raises moisture levels instantly, improves the look of skin texture and clarity, so you see clearer, more radiant-looking skin in just 2 weeks. (New Beauty 2010)

Ordinary would-be consumers of *SK*-*II Facial Treatment Essence* could not be expected to understand or differentiate between ‘miracle water’ and ‘Pitera™,’ for ‘miracle water’ refers to a metaphysical quality whose source cannot be ascertained through scientific method, whereas ‘Pitera™’ refers to a proprietary product ostensibly developed through scientific method, but owned exclusively by the company who makes it. Neverthless, insofar as the goal of the advertising is to convince the consumer of the anti-ageing promise of the product, the strategy of combining folk tales and scientific claims is very effective.

### Problems with Consumer Choice: ‘Scientific Consumerism’ and Selling Myths

However, this type of marketing strategy creates its own set of problems. The do-it-yourself health care model assumes an educated consumer receiving and critically evaluating different types of health care knowledge, on which basis they can make informed decisions about whether to purchase a given product or not. To illustrate, anti-ageing skin-whitening products such as *SK*-*II Facial Treatment Essence* that promise instantaneous and miraculous beauty transformations brought on by a combination of ‘miracle water’ and high-technology innovations are marketed under the label of cosmetics and not pharmaceuticals. As such, they are not regulated as drugs, and so their efficacy and health risks to consumers cannot be verified (Mire 2012). Moreover, since the product’s claims cannot be verified, the consumer often receives deliberately misleading and folksy information regarding the product, which is neither objective nor expert and exposes some of the fundamental problems with aggressive, consumer-direct marketing in the unregulated anti-ageing skin-whitening market. Rather, in the absence of objective and verifiable evidence that these products reverse or eliminate signs of ageing, it is a discursive use of the biomedicalization of ageing that sustains this practice and emerging lucrative market. Thus, the main drivers behind the proliferation of do-it-yourself anti-ageing skin whitening include strategic collaboration between enterprising health care professionals and corporations and fear of ageing in women. It is this strategic collaboration between health care experts and cosmetics and pharmaceutical corporations that supports the phenomenal rise of do-it-yourself anti-ageing skin-whitening ‘scientific consumerism’ (Mire 2012).

As has been already demonstrated, this section of the paper illustrates an increasing popularization of do-it-yourself anti-ageing skin-whitening scientific consumerism and draws extensively from the first high-end anti-ageing magazine *New Beauty* to reveal this emerging trend. As a quarterly upscale anti-ageing outlet for rich, famous, and image conscious consumers, each issue of *New Beauty* features a female Hollywood star, along with her favourite anti-ageing regimes. This is central to the philosophy of *New Beauty*. These celebrity profiles transmit the message that any woman can acquire ageless beauty like the featured Hollywood star if she chooses to select and consume the correct anti-ageing product. *New Beauty* differs from the average fashion or lifestyle magazine since it brings together high-technology anti-ageing skin-whitening products, female Hollywood stars, and listings of licensed *beauty docs™* who promote not only their expert knowledge, but in some cases, their own patented anti-ageing skin-whitening products directly to consumers. The advent of this new high-end anti-ageing ‘whiteness therapy’ is taking shape at the very moment when skin whitening is becoming a lucrative globalizing corporate economic activity (Ashikari 2005; Global Industry Analysts 2009a, b, c). As it was with commodification of soap in the colonial era, the contemporary anti-ageing skin-whitening market appeals to consumer desire for cleanliness, health, wellness and regenerative power of whiteness. Whether it is a colonial concern with racial degeneration or a current concern with the ageing bodies of women, whiteness is imbued with regenerative power. Thus, in the case of anti-ageing skin-whitening, images and idealized social constructs regarding beauty are being sold as inherent to the product. Therein lie the globalizing allure and dynamism of anti-ageing skin-whitening.

## Conclusion

This paper has demonstrated the extent to which the current phenomenon of do-it-yourself anti-ageing skin-whitening practice represents a re-articulation of Western colonial concerns with environmental pollution and racial degeneracy into an overwhelming concern with pigmentation pathology as a phenomenon of gendered vulnerability. Whether in colonial medicine or anti-ageing skin-whitening discourse, it is pigmented skin, that is, the site of pathology; healthy skin continues to be represented as that which is free from pigment. Anxiety over pigmentation makes a direct link between pigmented skin and pathological status. It is in this context that the current proliferation of anti-ageing whiteness therapy is therefore mobilised as means to remove these visible signs of ageing. However, in the anti-ageing whiteness therapy discourse, there is a dislocation and diffusion of the geographical locations of pollutions and sites of whiteness consumption. The current global flows of information, knowledge, people, goods, and services across national and international boundaries have reconfigured the very concepts of space and race (Appadurai 2001; Parr 2002). Thus, today, the old colonial concept of European North and threatening tropics has lost its meaning. As a result, exclusive anti-ageing health spas could be located in tropical oases, and the metropolises of the North could be sites of environmental pollution, degradation, and stress. Nonetheless, this paper has shown the residual presence of the colonial medical discourse and its relationship to health, disease, race, class, and gender in the current anti-ageing skin-whitening discourse. It is in this context that the social, ethical, health, and political implications of emerging anti-ageing skin-whitening must be comprehended and contested.
